# Social Q&A communities: A multi-factor study of the influence of users’ knowledge sharing behaviors

**DOI:** 10.3389/fpsyg.2022.967991

**Published:** 2022-09-29

**Authors:** Yi Wen, Xiaofang Yuan, Wenqin Li

**Affiliations:** Management School, Xi’an University of Science and Technology, Xi’an, China

**Keywords:** social Q&A community, knowledge sharing behaviors, perceived ease of use, perceived usefulness, perceived behavioral control, subjective norms, perceived security, perceived privacy

## Abstract

Recently, social Q&A communities have grown increasingly popular, serving as a primary platform for people to learn and share information. Nonetheless, fewer knowledge producers in these communities are significant than knowledge consumers. Thus, promoting users’ participation in knowledge sharing is a challenge for managers of social Q&A communities. Even though many scholars have studied factors influencing willingness to share knowledge, they tend to start with one theory and ignore the impact of several factors on behaviors. Thus, this manuscript presents a multi-factor model based on three dimensions of technology, cognition, and security to explore the effects of the six factors of perceived ease of use and perceived usefulness, perceived behavioral control and subjective norms, perceived security, and perceived privacy in terms of the three knowledge sharing methods of browsing including like and favorite, publishing and replying, and to compare users’ willingness to use the three knowledge sharing methods. A total of 482 questionnaires were collected online, and the hypotheses were tested and analyzed using structural equation modeling (SEM). According to the results, the factors affecting different sharing methods are not the same. Perceived behavioral control and perceived security can have a significant influence on their willingness to browse, users’ willingness to publish and reply to posts is significantly influenced by their perception of behavioral control and subjective norms, while perceived usefulness also affects their willingness to respond, it can be seen that cognition is the most important factor affecting users’ knowledge sharing among the three dimensions. In addition, users’ willingness to browse is significantly greater than their willingness to reply, and their willingness to post is the lowest.

## Introduction

In recent years, social networking services have become an important part of people’s lives as the Internet has become the primary source of knowledge and information (Glavas et al., 2019). The development of Q&A communities for the purpose of “knowledge sharing” has become an effective channel for the dissemination of information (Rosenbaum and Shachaf, 2010). Throughout the past several years, Q&A communities, which allow users to ask and answer their questions, have become one of the most important sources of online knowledge and information.

Through a social Q&A community, which is a hybrid between an encyclopedia and a fully functional Q&A community and its ability to deliver a more personalized search experience and results, making it superior to traditional information search methods (Zhe and Jansen, 2017), users can find quality content by focusing on topics, questions, answers, and other users. As a member of a social Q&A community, you will not only find appropriate answers, but you will also be able to meet more people with common interests. As one of the most popular knowledge sharing platforms, social Q&A communities also face several issues hampering their development, Q&A’s social activities are primarily handled by a small group of individuals in communities (Metzler et al., 2019), such as irrigation and free-riding within the community that seriously hinder the knowledge exchange between users (Lv and Li, 2022), active users in the community do not receive the expected reward resulting in a lower willingness to share their knowledge (Tu et al., 2022), the quality of user responses varies considerably and low-quality information is prevalent (Sheng et al., 2020). Currently, the two biggest challenges facing social Q&A communities are user loss and slowing active user growth, and the main reason for this is the lack of knowledge contribution from users within the community (Song et al., 2019). In order to improve users’ willingness to share knowledge, many scholars have studied the factors that influence users’ knowledge sharing. Knowledge sharing willingness is caused by multiple factors. There have been studies on the factors that influence knowledge sharing ability based on motivation theory, theory of planned behavior, social capital theory, and exchange theory, among others, factors like reward, self-efficacy, reputation, reciprocity, and information security can affect knowledge sharing willingness (Ortiz et al., 2017; Oliveira Jordao do Amaral and Kang, 2021; Nguyen et al., 2022). Furthermore, Armstrong A and Hagel J differentiated users into four categories: viewers, divers, contributors, and buyers (Armstrong and Hagel, 1998), later, some scholars categorize community users into five categories: inquirers, responders, divers and active users, and suggest to devise corresponding programs for each group (Zhang and Chu, 2020). Finally, in the light of knowledge sharing behaviors, scholars in previous virtual knowledge communities divided sharing of knowledge into three forms: browsing, initiating topics, and participating in discussions (Xu and Ye, 2011), In today’s social Q&A communities, members can like and favorite content while browsing. In this manuscript, liking and favoriting behaviors are classified as browsing behaviors, which means knowledge sharing methods include browsing (liking and favoriting), publishing and replying posts, these three sharing methods represent different degrees of user participation in Q&A communities. Participation of users in community interaction and active participation of users in knowledge sharing is the key to the prosperity of Q&A communities (Xu and Wang, 2007).

As a virtual community dedicated to solving users’ questions and answers, scholars have explored the influence of a variety of factors on willingness to share knowledge, but most of them have all studied individual factors from a particular theory without combining factors from different theories, and there are relatively few studies on the degree of influence of various factors on different ways of sharing knowledge. In this study, we construct a multidimensional model that examines technical factors (perceived usefulness, perceived ease of use), cognitive factors (perceived behavioral control, subjective norms), and safety factors (perceived security, perceived privacy) that influence knowledge sharing behaviors in social Q&A communities.

## Theoretical review

### Social Q&A community

Social Q&A communities have become increasingly popular in recent years, and their wide range of features make them stand out against traditional Q&A communities and search engines. In contrast to traditional Q&A communities, social Q&A communities combine social networking and Q&A functions, and the powerful social features facilitate easier and faster interaction between users, By sharing knowledge, users establish reciprocal norms that create a virtuous circle of knowledge sharing that benefits themselves and others (Wu and Korfiatis, 2014), users with common interests form their own interest circles and comply with the norms of interaction, forming a sense of belonging to the community (Bao and Han, 2019). In comparison with search engines, social Q&A communities provide a more inclusive platform, when they want first-hand information, different perspectives, and comparisons, users prefer social Q&A communities (Jeon and Rieh, 2013), a social Q&A community offers many benefits to users, including information contributed by members, insights shared by members, customizable information, and interaction with members to promote questions and answers generated in daily life, which has become a major challenge for search engines to address, search engines can only retrieve information from a database by matching keywords, which has a certain gap in terms of information quality and effectiveness (Lou et al., 2013). Social Q&A communities place more emphasis on knowledge and information exchange (Lou et al., 2013), besides being knowledge consumers, users are knowledge producers and disseminators, as a member of the social Q&A community, you can read and browse content posted by other members, and you can also publish your own questions, answers, and ideas, as well as communicate with other members, including asking questions, inviting answers, following, and commenting. The quality of information is evaluated according to the number of likes, favorites, views, and other factors, and the best information will be promoted first. Users cannot always choose the most appropriate answer in a social Q&A community flooded with information streams (Elalfy et al., 2018), therefore, a number of scholars have proposed optimizations to the information quality ranking results in social Q&A communities, Alam developed a reputation model based on dynamic points for users, referring to the retrieval of high-quality information for users to improve user satisfaction (Alam et al., 2016). Figueroa generates and identifies search strings directly from the questions asked, allowing efficient retrieval of relevant questions in the community knowledge base (Figueroa, 2017), afterward, Palomera and Figueroa (2017) use semi-supervised machine learning to improve retrieval accuracy. To help users make more informed decisions, Chi et al. (2019) propose a three-stage supernetwork-based model.

### Knowledge sharing

While social Q&A communities offer great advantages, they face the growing challenge of encouraging and maintaining ongoing user engagement in knowledge sharing (Chen, 2020). Sharing knowledge is an important learning activity and socializing behavior for learners, as well as a prerequisite for the creation of knowledge and activation of knowledge communities online (Li and Wang, 2021a). Users are mostly active as receivers in the community, while those who create are few (Cai and Yue, 2018). Many online Q&A communities try to stimulate knowledge sharing by implementing gamification mechanisms (Chen et al., 2022). It is important to note, however, that many factors influence users’ willingness to participate in knowledge sharing (Tausczik and Huang, 2020), this is related to the needs and intentions of the user (Shwartz-Asher et al., 2020). Based on motivation theory, willingness to share knowledge is affected by internal and external motivation, and factors such as utilitarian motivation, hedonic motivation, and self-efficacy influence knowledge sharing (Liao et al., 2013). Users are more likely to share knowledge when they receive reciprocal expectations, which leads to increased self-efficacy, as well as increased willingness to continue sharing (Cheung et al., 2013), the virtual points earned by submitting knowledge to the Q&A community are used as an extrinsic motivation, and they can be redeemed for reward (Zhao et al., 2016). It is also possible for the attitude of the organization toward knowledge sharing to influence people’s beliefs about sharing knowledge or hiding it (Pereira and Mohiya, 2021). Additionally, sharing knowledge online also involves a degree of risk, thus, some scholars analyze the game using transaction costs and social exchange theory to examine willingness to share knowledge and insecure behaviors such as privacy leakage (Fatima et al., 2019; Li and Kang, 2019), individual security awareness and information trustworthiness both have an impact on knowledge sharing behavior (Ortiz et al., 2017), risks and benefits are then weighed to determine knowledge sharing behavior (Tsai et al., 2013). In social Q&A communities, user behavior can also be influenced by interpersonal relationships, Ma and Chan (2014) demonstrate that both altruism and perceived online relationships significantly influence user knowledge sharing using an interpersonal model. Further divisions have been made between poster and lurker users in Q&A communities, implying that posters and lurkers have different levels of responsibility for sharing knowledge in social Q&A communities, for posters, knowledge self-efficacy is the most influential factor, while for lurkers, and resource availability is the most important (Shin-Yuan et al., 2015).

## Research hypothesis and model

**FIGURE 1:**
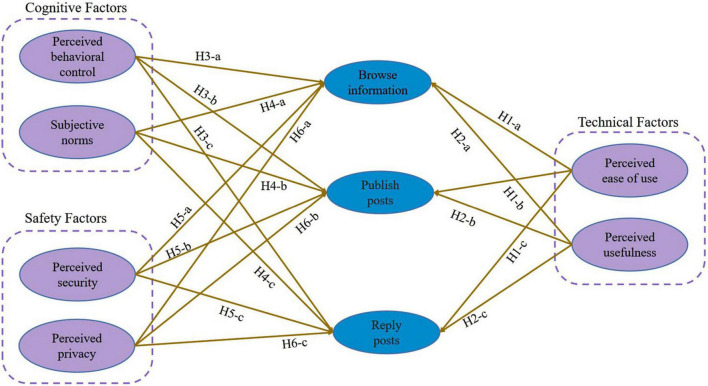
Research model.

In summary various factors can affect users’ willingness to share knowledge in social Q&A communities. For example, the degree of knowledge sharing between knowledge sharers, the sharing environment, the sharing technology, and the shared knowledge itself are all factors that can influence the degree of knowledge sharing (Li and Wang, 2021b). Consequently, this manuscript selects perceived ease of use and perceived usefulness as technical factors from the technology acceptance mode (Davis, 1989); Based on Ajzen’s theory of planned behavior (Ajzen, 1991), attitudes, subjective norms, and perceived behavioral control can determine willingness. Using the theory of planned behavior, the manuscript selected perceived behavioral control and subjective norms as cognitive factors; In accordance with social exchange theory (Cropanzano and Mitchell, 2005), perceived security and perceived privacy are taken as being the cost of security in the knowledge sharing process, with the six factors, we construct a model and explore how they affect the three behaviors of knowledge sharing, research model see Figure 1.

### Hypotheses of perceived usefulness, perceived ease of use, and knowledge sharing behaviors

According to Technology Acceptance Model (TAM), perceived usefulness and perceived ease of use are the two primary factors that determine users’ attitudes and willingness to accept. Considering the definition and characteristics of social Q&A communities, in this article the perceived ease of use refers to the time and effort it takes for users to be able to learn and become proficient at harvesting knowledge from social Q&A communities, perceived usefulness means the actual effect of the answers users get in the social Q&A community. Perceived ease of use increases the speed of knowledge exchange between users and saves time in knowledge exchange, If users find a particular social Q&A community easy to use, they are more likely to take advantage of the virtual community to share knowledge (Li, 2013). Similarly, when users find the platform to be useful, they will feel inclined to share knowledge based on their satisfaction with the platform’s feature set. If users are satisfied with the knowledge sharing platform, they will show a strong willingness to use it (Pang et al., 2020). According to the discussion above, the following hypotheses can be made:

*H1-a: Perceived ease of use can positively influence users’ browsing behavior.*

*H1-b: Perceived ease of use can positively influence users’ publishing posts behavior.*

*H1-c: Perceived ease of use can positively influence users’ replying posts behavior.*

*H2-a: Perceived usefulness can positively influence users’ browsing behavior.*

*H2-b: Perceived usefulness can positively influence users’ publishing posts behavior.*

*H2-c: Perceived usefulness can positively influence users’ browsing behavior.*

### Hypotheses of perceived behavioral control, subjective norms, and knowledge sharing behaviors

Subjective norms and perceived behavioral control are important factors influencing individuals’ behavioral intentions in the theory of planned behavior. Feng et al. (2021) demonstrated that the theory of planned behavior has strong predictive power for willingness to share knowledge, individuals’ willingness to share knowledge can be influenced by perceived behavioral control and subjective norms (Zheng et al., 2014). An individual’s perceived behavioral control refers to the extent to which he or she feels comfortable performing a particular behavior given their past experiences and expected barriers. The subjective norms are the social expectations that users feel when deciding whether to take part in knowledge sharing, and it indicate the social pressure on individuals to perform a certain behavior. In his study, Park et al. (2014) emphasized that willingness and ability to share are both important factors in sharing behaviors. According to the discussion above, the following hypotheses can be made:

*H3-a: Perceived behavioral control can positively influence user’s browsing behavior.*

*H3-b: Perceived behavioral control can positively influence users’ publishing posts behavior.*

*H3-c: Perceived behavioral control can positively influence users’ replying posts behavior.*

*H4-a: Subjective norms can positively influence user’s browsing behavior.*

*H4-b: Subjective norms can positively influence users’ publishing posts behavior.*

*H4-c: Subjective norms can positively influence users’ replying posts behavior.*

### Hypotheses of perceived security, perceived privacy, and knowledge sharing behaviors

Social Q&A communities require users to create accounts in order to browse, post, answer, favorite, and follow, etc. Therefore, users must be willing to share their private or public information, including username, email address, education details, and life status, etc. (Tian et al., 2018). Similar information can also be delivered by platforms based on the digital behavior of users, including the information explored and the time spent. While users enjoy the personalized services provided by platforms, they also face the risk of having their personal information exposed and abused (Luo, 2016). In Mayer’s definition, security is defined as the result of trust between two parties, where trust has a high perceived value for the trustor (Mayer et al., 1995), As companies implement different security measures to protect hardware, software, company data, user data, and users feel more secure and confident when they know that the platform is vigilant and taking action against potential threats, it is important that each company has its own security strategy. When users perceive a decreased risk of privacy breaches, they are more likely to share and the information they share is more accurate (Mattison Thompson and Siamagka, 2022). As well as, perceived security can have a significant impact on sharing behavior (Zhang and Zhou, 2019). According to the discussion above, the following hypotheses can be made:

*H5-a: Perceived security can positively influence user’s browsing behavior.*

*H5-b: Perceived security can positively influence users’ publishing posts behavior.*

*H5-c: Perceived security can positively influence users’ replying posts behavior.*

*H6-a: Perceived privacy can negatively influence user’s browsing behavior.*

*H6-b: Perceived privacy can negatively influence user’s publishing posts behavior.*

*H6-c: Perceived privacy can negatively influence user’s replying posts behavior.*

## Research methodology

### Questionnaire design

For reliability and validity, the measurement tool used in this study was based on well-established scales at home and abroad (Cheung and Lee, 2001; Hsu et al., 2007; Lin, 2007; Cheon et al., 2012; Tamjidyamcholo et al., 2014), which were then modified and refined following the research’s content. In the first part of the questionnaire, users are asked for basic personal information (gender, age, and education level) as well as if they have used the social Q&A community; The second section examines factors that influence the knowledge sharing behaviors found in social Q&A communities, with a total of 72 questions, six factors were examined for all three types of knowledge sharing behaviors. The questionnaire is designed on the basis of the seven-point Likert scale, with “1” indicating “strongly disagree” and “7” indicating “strong agree.” Detailed information on the measurement indexes and references adopted in this questionnaire can be found in Supplementary material.

### Data collection and sample statistics

The questionnaire was primarily collected from Chinese users who have joined social Q&A communities, and it was distributed and collected from March 20, 2022 to May 5, 2022. We collected a total of 482 questionnaires online, removing some that took less time and contradicted each other since they might indicate that respondents did not read carefully, the number of valid questionnaires browsed, published, and replied were 426, 380, and 439, respectively, with an average efficiency rate of 86%. A descriptive analysis was conducted on questionnaire responses through SPSS 23.0, the results are shown as an example of data collected by browsing questionnaires, 42% of males and 58% of females were equally represented, with females representing a slightly higher percentage. In terms of age, the 21 to 25-year-old group represents more than half with 60%, followed by the group of less than 20-year-olds with 33%, indicating that the respondents to this questionnaire tend to be younger. Among educational attainment, the largest percentage is undergraduate (59%), followed by high school, and below (18%). See Table 1.

**TABLE 1:** Demographic frequency analysis.

Variables	Items	Frequency	Percentage (%)
Gender	Male	176	42
	Female	250	58
Age	Below 20	140	33
	21–25	260	61
	26–30	21	5
	31–40	5	1
Academic degree	High school or below	75	18
	Junior college	29	7
	Undergraduate	250	59
	Master	69	16
	Doctor	3	1

## Data analysis and research result

### Reliability and validity

In order to verify the reliability of the questionnaire, SPSS 23.0 and AMOS 23.0 were used to analyze the reliability and validity of the questionnaire data. Cronbach’s alpha is used to measure reliability, when this coefficient reaches between 0.7 and 0.8, the reliability of the question item is considered good, and when it reaches 0.8, the reliability of the question item is considered excellent. To verify the validity of the questionnaire, confirmatory factor analysis (CFA) was performed and the combined reliability (CR) of each latent variable was greater than 0.7, indicating high internal consistency of the model variables. When factor loadings are greater than 0.6 and average variance extracted (AVE) are greater than 0.5, the model is considered to have convergent validity, Among the questions in the perceived privacy dimension, one did not reach the standard factor loading, so it was eliminated, This questionnaire’s reliability and validity after adjustment are shown in Table 2, with factor loadings greater than 0.7 and AVE values greater than 0.6, CR and Cronbach’s alpha values are both greater than 0.8, indicating high reliability and good convergence of internal consistency. The discriminant validity can be examined by examining whether the AVE square root of latent variables is greater than their correlation coefficients, as shown in Table 3, the model has good discriminant validity.

**TABLE 2:** Reliability and validity analysis.

Construct	Indicators	Factor loading	SMC	AVE	CR	Cronbach’s α
Perceived ease of use (PEU)	PEU1	0.858	0.736	0.754	0.925	0.924
	PEU2	0.822	0.676			
	PEU3	0.874	0.763			
	PEU4	0.917	0.790			
Perceived usefulness (PU)	PU1	0.795	0.628	0.653	0.883	0.883
	PU2	0.857	0.731			
	PU3	0.764	0.577			
	PU4	0.823	0.677			
Perceived behavioral control (PBC)	PBC1	0.742	0.559	0.602	0.818	0.816
	PBC2	0.785	0.613			
	PBC3	0.792	0.628			
Subjective norms (SN)	SN1	0.754	0.568	0.586	0.809	0.811
	SN2	0.768	0.590			
	SN3	0.774	0.599			
Perceived security (PS)	PS1	0.864	0.747	0.767	0.908	0.908
	PS2	0.866	0.751			
	PS3	0.896	0.803			
Perceived privacy (PP)	PP1	0.856	0.738	0.816	0.898	0.896
	PP2	0.947	0.899			
Browse information (BI)	BI1	0.712	0.669	0.709	0.907	0.872
	BI2	0.844	0.780			
	BI3	0.792	0.666			
	BI4	0.829	0.721			

CR, composite reliability; AVE, average variance extraction; SMC, squared multiple correlation. The data are all based on the willingness to browse information. Supplementary contains data related to reply and publish.

**TABLE 3:** Discriminant validity analysis.

Construct	PEU	PU	PBC	SN	PS	PP	BI
Perceived ease of use (PEU)	1						
Perceived usefulness (PU)	0.686	1					
Perceived behavioral control (PBC)	0.669	0.758	1				
Subjective norms (SN)	0.411	0.535	0.643	1			
Perceived security (PS)	0.077	0.229	0.281	0.372	1		
Perceived privacy (PP)	0.021	0.138	0.169	0.314	0.866	1	
Browse information (BI)	0.483	0.518	0.625	0.447	0.142	0.132	1
AVE square root	0.868	0.808	0.775	0.765	0.876	0.903	0.842

### Structural model equation validation

AMOS 23.0 was used to evaluate the model for goodness-of-fit, and the results and standard values of goodness-of-fit for the structural and measurement models can be seen in Table 4. As can be seen in the Table 4, except for the GFI value which is slightly lower than 0.9, all other indicators meet the fitness criteria, and the calculation results are acceptable, indicating that the model has overall good goodness of fit.

**TABLE 4:** The study of the model fitting index and proposed values.

Evaluation indicators	Structural model	Measurement model	Standard value
χ^2^/*df*	2.779	2.223	<3
RMSEA	0.065	0.054	<0.08
NFI	0.916	0.933	>0.9
IFI	0.945	0.962	>0.9
TLI	0.933	0.954	>0.9
CFI	0.944	0.962	>0.9
GFI	0.893	0.912	>0.9
AGFI	0.859	0.883	>0.8

RMSEA, root mean square error of approximation; NFI, normed fit index; IFI, incremental fit index; TLI, tucker-lewis coefficient; CFI, comparative fit index; GFI, goodness of fit index; AGFI, adjusted goodness of fit index

### Hypothesis testing

**FIGURE 2:**
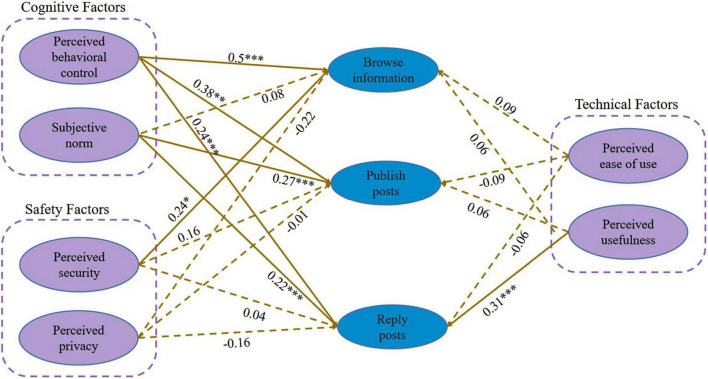
Result of the research model. ***p < 0.001, **p < 0.01, *p < 0.05.

The model path coefficients and hypothesis testing results are shown in Table 5. Perceived behavioral control (*p* < 0.001), and perceived security (*p* < 0.05) had a significant influence on knowledge browsing, supporting hypotheses H3-a, H5-a; Perceived behavioral control (*p* < 0.001) and subjective norms (*p* < 0.001) has a significantly positive influence on publishing posts, supporting hypothesis H3-b and H4-b; Perceived usefulness (*p* < 0.001), subjective norms (*p* < 0.05), and perceived behavioral control (*p* < 0.001) significantly positively influenced replying behavior, supporting hypotheses H2-c, H3-c, and H4-c. In terms of technical, cognitive, security dimensions, some cognitive factors (perceived behavioral control), and security factors (perceived security) have significant effects on knowledge browsing behavior, however, it was found that technological factors (perceived ease of use, perceived usefulness) did not have a significant influence on knowledge browsing behavior. Among the factors influencing publishing posts behavior, factors in the cognitive dimension were significant, but factors in the technical and security dimensions did not appear to be significant. Regarding reposting behavior, security factors are not significantly influencing reposting behavior, H5-c and H6-c are not supported in this study sample, and factors in some technical dimensions (perceived usefulness) and factors related to cognition are significantly influencing reposting behavior. In summary, part of the research hypotheses are supported, as shown in Table 5 and Figure 2.

**TABLE 5:** Result of hypothesis analysis.

Paths	Estimate	S.E.	C.R.	*P*	Results
H1-a: Perceived ease of use → browse information	0.089	0.068	1.228	0.22	Unsupported
H2-a: Perceived usefulness → browse information	0.062	0.087	0.695	0.487	Unsupported
H3-a: Perceived behavioral control → browse information	0.499	0.12	4.374	***	Supported
H4-a: Subjective norms → browse information	0.076	0.067	1.024	0.306	Unsupported
H5-a: Perceived security → browse information	0.236	0.063	1.967	0.047	Supported
H6-a: Perceived privacy → browse information	–0.218	0.064	–1.888	0.059	Unsupported
H1-b: Perceived ease of use → publish posts	–0.089	0.076	–1.275	0.202	Unsupported
H2-b: Perceived usefulness → publish posts	0.056	0.111	0.63	0.529	Unsupported
H3-b: Perceived behavioral control → publish posts	0.38	0.128	3.665	***	Supported
H4-b: Subjective norms → publish posts	0.274	0.092	3.695	***	Supported
H5-b: Perceived security → publish posts	0.159	0.113	1.039	0.299	Unsupported
H6-b: Perceived privacy → publish posts	0.006	0.105	–0.038	0.969	Unsupported
H1-c: Perceived ease of use → reply posts	–0.064	0.079	–0.923	0.356	Unsupported
H2-c: Perceived usefulness → reply posts	0.308	0.077	4.204	***	Supported
H3-c: Perceived behavioral control → reply posts	0.244	0.112	2.506	0.012	Supported
H4-c: Subjective norms → reply posts	0.22	0.059	3.646	***	Supported
H5-c: Perceived security → reply posts	0.038	0.054	0.435	0.663	Unsupported
H6-c: Perceived privacy → reply posts	–0.156	0.052	–1.844	0.065	Unsupported

***p < 0.001.

**FIGURE 3:**
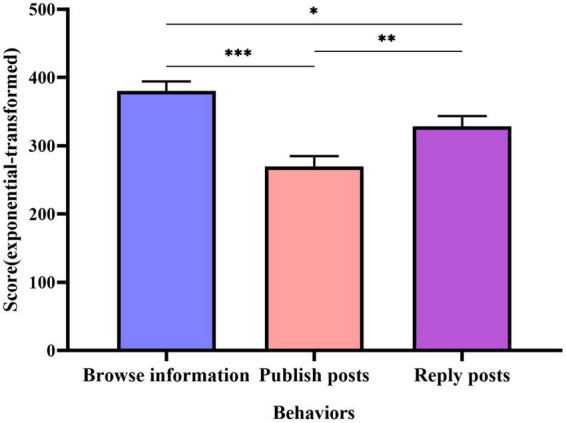
LSD *post-hoc* comparison of the main effects of categories and scores. ***p < 0.001, **p < 0.01, *p < 0.05.

An analysis of variance (ANOVA) was used to analyze the willingness to share scores for the three behaviors, after exponential-transforming the scores of the three shared willingness scores to satisfy the homogeneity of variance test, and the results revealed significant main effects for all three behaviors: *F*(2,1242) = 13.453, *p* < 0.001, following the least-significant difference (LSD) *post-hoc* comparison, there was a significant difference (*p* < 0.001) between willingness to browse (*M* = 380.23, SD = 294.77) and willingness to post (*M* = 269.79, SD = 295.28), between willingness to browse and reply (*M* = 328.57, SD = 313.74; *p* = 0.012 < 0.05), and between willingness to publish and reply (*p* = 0.006 < 0.01). Indicating that users’ willingness to share knowledge is different for the three knowledge sharing behaviors, with the willingness to browse information having significantly higher scores than the willingness to publish and reply, and the willingness to reply having significantly higher scores than posting. See Figure 3.

## Discussion and conclusion

### Discussion

As a result of the Internet’s development, social Q&A communities are becoming a popular platform for online interactions and knowledge sharing. It is important for all social Q&A communities to think about getting more active users, creating a good atmosphere for knowledge sharing, and encouraging more users to take part in knowledge sharing. In the context, this study identified six factors as influencing factors for browsing information, posting, and replying in three dimensions, namely technology, cognition, and safety, and the differences among the various factors on knowledge sharing behaviors were explored further.

The results of the validation analysis in this study demonstrated that perceived behavioral control and perceived security had a positive and significant influence on browsing information in social Q&A communities, with path coefficients of 0.50 and 0.24 for perceived behavioral control, perceived security, and browsing information, it can be concluded that perceived behavioral control is the predominant consideration for users when browsing information in social Q&A communities. Perceived behavioral control is the perception of the difficulty of performing the behavior, indicating that for the user, browsing information meets their abilities and expectations, which is consistent with the findings of Nader, Hashmi (Safa and Von Solms, 2016; Hashmi et al., 2021). Browsing as a basic way for users to participate in social Q&A communities, and intent to use when users feel safe with online communities (Ha and Pan, 2018), more users are inclined to visit online platforms that pay attention to the protection of their personal information, such as their ID, account information, and phone number, Zhihu, for instance, offers a browsing-only mode in which no personal information is collected, allowing users to get an initial experience of the platform, and thus perceived security influences users’ willingness to browse, a finding similar to some other researchers (Klobas et al., 2019; Maqableh et al., 2021). Users’ publishing posts behavior is positively influenced by perceived behavioral control and subjective norms, with model path coefficients of 0.38 and 0.274, respectively, and there is no significant difference between the two effects, demonstrating that the cognitive factor is the most significant influence on publishing behavior. Publishing posts behavior has higher social Q&A community engagement than simply browsing for information, publishing can be posting questions, ideas, videos, columns, and articles, etc. When users think they are capable and willing to post for information sharing, or when they are invited by some netizens for knowledge sharing, the pressure from outside can promote users’ posting behavior, and this conclusion is consistent with the conclusion of some researchers (Xiong and Xia, 2014). In other words, if a user feels more people are eager to learn from him, he is more likely to contribute. In the case of replying behavior, in addition to perceived behavioral control and subjective norms having a positive and significant effect on replying, perceived usefulness also has a significant influence on replying behavior with their paths of 0.24, 0.22, and 0.31, it was found that perceived usefulness had a higher influence on replying behavior than the other two, Sussman and Siegal (2003) asserted that perceived usefulness plays an important role in information adoption, Park et al. (2014) argued that investors will tend to seek information in communities with higher levels of usefulness, A reply can be a further exploration of information sharing, either by responding to other posts or by expressing gratitude to the poster. From the results of the analysis, it can be concluded that users are more likely to reply when they find the information valuable. In fact, there is a relationship between the three behaviors themselves, when users are influenced by perceived security to participate in a social Q&A community, browsing for information is the first step in their participation in the community, replying and publishing posts are higher degree of participative behaviors. Due to a demand for knowledge information, users may pay less attention to security factors. In both cases, perceived security and perceived privacy are not significant influences on publishing and replying to posts. The results in Table 5 show that perceived privacy has no significant impact on any of the three knowledge sharing behaviors, which is inconsistent with the findings of some researchers (Zhu et al., 2020; Grande et al., 2022).

Moreover, we used one-way ANOVA to compare the willingness to browse, publish, and reply to posts, as shown in Figure 3, in this study, the willingness to browse scored highest and the willingness to publish posts ranked lowest, and there were significant differences between the three behaviors. It can be inferred that when users participate in social Q&A communities, they are more willing to browse and search for information, which is the quickest way to find out what others think by checking the comment section while searching for information, or, if they need more information, they can reply to posters or discuss with other responders in order to gain a complete understanding of the topic. The relatively low willingness to publish posts score may be the result of the fact that most users believe they are unable to share knowledge and information, or that it requires spending more effort than browsing and responding to posts, therefore, they do not prefer to participate in knowledge sharing by posting in social Q&A communities.

### Conclusion

Within social Q&A communities, users can participate in knowledge sharing in a variety of ways, and users’ willingness to use different sharing methods varies. This study classifies knowledge sharing behaviors, from technical, cognitive, and security perspectives, this study examined how perceived ease of use, perceived usefulness, perceived behavioral control, subjective norms, perceived security, and perceived privacy influence the behaviors of knowledge sharing in social Q&A communities, and the experiment and analysis have academic implications for improving users’ engagement and willingness to share knowledge in social Q&A communities. Based on the results of our study, the following recommendations are provided.

As the initial behavior of users joining a social Q&A community, users may place more importance on whether they can adapt to the platform and the platform’s protection of user security. Administrators should pay more attention to the platform’s security protection mechanism, provide more reliable security protection for users, enhance users’ perceived security, and lay the foundation for subsequent users’ in-depth use of the platform.

Publishing and replying to posts as the most important behaviors in the social Q&A community, whether it is publishing questions that resonate with platform users, inspiring experienced people to reply, or publishing articles that produce high-quality knowledge sharing articles, all have a positive effect on the platform to create a knowledge sharing atmosphere. In the analysis, both individual perceived behavioral control and subjective norms have a significant positive impact on publishing posts behavior, which also requires platform administrators to improve the posting mechanism so that users are more willing to publish posts when they feel they can publish and share knowledge according to their own wishes without the restrictions and operations of the website; Meanwhile, the platform should strengthen the interaction between users and the platform, users and users (such as fans), and enhance the willingness to communicate between users.

In addition to perceived behavioral control and subjective norms, perceived usefulness also has a significant positive impact on users’ replying behavior. Usefulness requires access to high quality information, which is a precondition for users to engage with reposts, and social Q&A platforms should continually optimize their information quality and improve their information search mechanisms to enable users to find more high-quality content, which is both beneficial for retaining users and attracting new ones.

With the development of the Internet, people are becoming more and more familiar with online platforms, and each online platform is committed to providing better services to users. Following the results of the previous analysis, it is evident that technical factors and safety factors are no longer the primary factors affecting people’s knowledge sharing, compared to users’ cognitive level, perceived behavioral control, and subjective norms, which are still the main factors affecting people’s knowledge sharing, so how to improve users’ cognitive level and promote their willingness to share may be the first issue that managers of social Q&A communities need to consider.

## Limitations

Although the results of this study have some practical implications for guiding the promotion of user engagement in social Q&A communities, there are some limitations and shortcomings. Firstly, the user groups who completed the questionnaire were not evenly distributed, and the users in this experiment were younger, mostly at the stage below 25 years old, and the younger group may not be sensitive to perceived privacy, which may be the reason why this factor of perceived privacy did not show significant differences among the three knowledge sharing methods, Hence, it is recommended that more statistics regarding users of different age groups are obtained. Furthermore, the questionnaire design does not provide stats on users’ reliance on and frequency of using social Q&A communities, so the user profile is incomplete, and should be included in future studies. Secondly, only certain factors from the technical, cognitive, and security dimensions are taken into account in this research, but other factors with added research value could be added in order to enrich the research model further, in order to further enrich the depth of the study, it would be more meaningful to continue to explore whether these factors directly or indirectly influence users’ knowledge sharing behavior and whether they can be used as moderating variables to influence users’ behavior in the future. Finally, with the development of social Q&A communities, the concept of paying for knowledge has gradually become more widely accepted by most people, and some users are already prepared to pay for better quality information, so the mechanism for sharing knowledge goes beyond browsing, publishing and replying, includes paid Q&A, invited answers, and so forth. It would be worthwhile to investigate which factors will affect the users’ payment behavior in future research.

## Data availability statement

The original contributions presented in this study are included in the article/Supplementary material, further inquiries can be directed to the corresponding author.

## Ethics statement

Ethical review and approval was not required for the study on human participants in accordance with the local legislation and institutional requirements. Written informed consent from the patients/participants or patients/participants legal guardian/next of kin was not required to participate in this study in accordance with the national legislation and the institutional requirements.

## Author contributions

All authors listed have made a substantial, direct, and intellectual contribution to the work, and approved the submitted version.
