# Supplementary material for: Social Q&A communities: A multi-factor study of the influence of users’ knowledge sharing behaviors
Source: Front Psychol. 2022 Sep 29;13:967991. doi: 10.3389/fpsyg.2022.967991 (PMC9556872; doi:10.3389/fpsyg.2022.967991)
Supplement: Supplementary file 1 [file Data_Sheet_1.PDF]

Supplementary

Part Ⅰ. Survey on browsing behavior				
construct	Measure	Loading	C.R	AVE
Perceived ease of use (Cronbach' α = 0.924)				
PEUI	Browsing (liking, favoriting) information is easy for me.	0.858	0.925	0.754
PEU2	Browsing (liking, favoriting) information is clear and understandable.	0.822		
PEU3	It would be easy for me to become skillful at browsing (liking, favoriting) information.	0.874		
PEU4	Overall, browsing (liking, favoriting) information in social Q&A community is easy for me.	0.917		
Perceived usefulness (Cronbach' α = 0.883)				
PU1	Browsing (liking, favoriting) information in social Q&A communities enhances my ability to get information.	0.795	0.883	0.653
PU2	Browsing (liking, favoriting) information in social Q&A communities enables me to share knowledge with my friends.	0.857		
PU3	Browsing (liking, favoriting) information in social Q&A communities helps satisfy my information needs.	0.764		
PU4	Overall, browsing (liking, favoriting) information in social Q&A communities meets my needs.	0.823		
Perceived behavioral control (Cronbach' α = 0.816)				
PBC1	Browsing (liking, favoriting) information in social Q&A communities is easy and enjoyable.	0.742	0.818	0.6
PBC2	I would like to browse (like, favoriting) information in social Q&A communities to facilitate knowledge sharing.	0.785		
PBC3	I have the ability to browse (like, favoriting) information I need in social Q&A communities.	0.792		
Subjective norms (Cronbach' α = 0.81)				
SN1	Users in social Q&A communities want me to browse (like, favorite) information	0.754	0.809	0.586
SN2	Most people who follow me would like me to like or favorite useful information in social Q&A communities.	0.768		
SN3	The community administrator would like me to browse (like, favorite) information in social Q&A communities.	0.774		
Perceived security (Cronbach' α =0.908)				
PS1	When browsing (liking, favoriting) in social Q&A communities, I think the platform is able to protect my account information.	0.864	0.908	0.767
PS2	Personal information will not be intercepted by third-party platforms when browsing (liking, favoriting) in social Q&A communities.	0.866		
PS3	It is safe to browse (like, favorite) in social Q&A communities.	0.896		
Perceived privacy (Cronbach' α = 0.896)				
PP1	I would be concerned about personal privacy when I browse (like, favorite) information in social Q&A communities.			
PP2	Platforms will not leak my personal information to third-party platforms when I browse (like, favorite) in social Q&A communities.	0.856	0.898	0.816
PP3	The privacy protection of the platform is reliable when I browse (like, favorite) in social Q&A communities.	0.947		
Knowledge Sharing Behavior (Cronbach' α = 0.872)				
KSB1	I frequently share with my network of friends the information that I browse (like, favorite) in social Q&A communities	0.712	0.907	0.709
KSB2	I frequently browse (like, favorite) information in social Q&A communities.	0.844		
KSB3	I usually spend a lot time browsing (liking, favoriting) information in social Q&A communities.	0.792		
KSB4	When discussing a hot issue, I will browse (like, favorite) information in social Q&A communities.	0.829		

PARTⅡ. Survey on publishing post behavior				
construct	Measure	Loading	C.R	AVE
Perceived ease of use (Cronbach' α = 0.897 ）				
PEU1	Publishing posts is easy for me.	0.751	0.898	0.690

PEU2	Publishing posts process is clear and understandable.	0.779		
PEU3	It would be easy for me to become skillful at publishing posts.	0.868		
PEU4	Overall, publishing posts in social Q&A community is easy.	0.913		
Perceived usefulness (Cronbach’ α = 0.890)				
PU1	Publishing posts in social Q&A communities enhances my ability to get information.	0.832	0.892	0.674
PU2	Publishing posts in social Q&A communities enables me to share knowledge with my friends.	0.851		
PU3	Publishing posts in social Q&A communities helps satisfy my information needs.	0.744		
PU4	Overall, Publishing posts in social Q&A communities meets my needs.	0.853		
Perceive behavioral control (Cronbach’ α = 0.871)				
PBC1	Publishing posts in social Q&A communities is easy and enjoyable.	0.873	0.883	0.717
PBC2	I would like to publish posts in social Q&A communities to facilitate knowledge sharing.	0.865		
PBC3	I have the ability to publish posts I need in social Q&A communities.	0.801		
Subjective norms (Cronbach’ α = 0.822)				
SN1	Users who focus on the same field in social Q&A communities want me to publish posts.	0.772	0.826	0.614
SN2	Most people who follow me would like me to publish posts in social Q&A communities.	0.856		
SN3	The community administrator would like me to publish posts in social Q&A communities.	0.716		
Perceived security (Cronbach’ α = 0.941)				
PS1	When publishing posts in social Q&A communities, I think the platform is able to protect my account information.	0.895	0.942	0.845
PS2	Personal information will not be intercepted by third-party platforms when publishing posts in social Q&A communities.	0.949		
PS3	It is safe to publish posts in social Q&A communities.	0.913		
Perceived privacy (Cronbach’ α = 0.927)				
PP1	I feel secure about personal privacy when I publish posts in social Q&A communities.			
PP2	Platforms will not leak my personal information to third-party platforms when I publish posts in social Q&A communities.	0.915	0.938	0.883
PP3	The privacy protection of the platform is reliable when I publish posts in social Q&A communities.	0.964		
Knowledge Sharing Behavior (Cronbach’ α = 0.937)				
KSB1	I frequently share with friends the information that I get form publishing posts in social Q&A communities.	0.907	0.937	0.79
KSB2	I frequently publish posts in social Q&A communities.	0.925		
KSB3	I am usually willing to spend a lot time publishing posts in social Q&A communities.	0.918		
KSB4	When discussing a hot issue, I will publish posts in social Q&A communities.	0.802		

PART Ⅲ. Survey on replying posts behavior				
construct	Measure	Loading	C.R	AVE
Perceived ease of use (Cronbach’ α = 0.933)				
PEU1	Replying posts is easy for me.	0.843	0.933	0.778
PEU2	Replying posts process is clear and understandable.	0.886		
PEU3	It would be easy for me to become skillful at replying posts.	0.897		
PEU4	Overall, replying posts in social Q&A community is easy for me.	0.901		
Perceived usefulness (Cronbach’ α = 0.904)				
PU1	Replying posts in social Q&A communities enhances my ability to get information.	0.809	0.906	0.707
PU2	Replying posts in social Q&A communities enables me to share knowledge with my friends.	0.872		
PU3	Replying posts in social Q&A communities helps satisfy my information needs.	0.770		

PU4	Overall, Replying posts in social Q&A communities meets my needs.	0.905		
Perceived behavioral control (Cronbach’ α = 0.854)				
PBC1	Replying posts in social Q&A communities is easy and enjoyable.	0.765	0.857	0.667
PBC2	I would like to reply posts in social Q&A communities to facilitate knowledge sharing.	0.837		
construct	Measure	Loading		
PBC3	I have the ability to reply posts I need in social Q&A communities.	0.845		
Subjective norms (Cronbach’ α = 0.869)				
SN1	Users who focus on the same field in social Q&A communities want me to reply posts.	0.864	0.870	0.690
SN2	Most people who follow me would like me to reply posts in social Q&A communities.	0.816		
SN3	The community administrator would like me to reply posts in social Q&A communities.	0.811		
Perceived security (Cronbach’ α = 0.954)				
PS1	When replying posts in social Q&A communities, I think the platform is able to protect my account information.	0.914	0.954	0.874
PS2	Personal information will not be intercepted by third-party platforms when replying posts in social Q&A communities.	0.957		
PS3	It is safe to reply posts in social Q&A communities.	0.934		
Perceived privacy (Cronbach’ α = 0.932)				
PP1	I feel secure about personal privacy when I reply posts in social Q&A communities.			
PP2	Platforms will not leak my personal information to third-party platforms when I reply posts in social Q&A communities.	0.959	0.927	0.962
PP3	The privacy protection of the platform is reliable when I reply posts in social Q&A communities.	0.967		
Knowledge Sharing behavior (Cronbach’ α = 0.922)				
KSB1	I frequently share with friends the information that I get form publishing posts in social Q&A communities.	0.896	0.752	0.924
KSB2	I frequently reply posts in social Q&A communities.	0.863		
KSB3	I am usually willing to spend a lot time replying posts in social Q&A communities.	0.898		
KSB4	When discussing a hot issue, I will reply posts in social Q&A communities.	0.808		
